# A Self-Powered Vector Angle/Displacement Sensor Based on Triboelectric Nanogenerator

**DOI:** 10.3390/mi12030231

**Published:** 2021-02-25

**Authors:** Chengyu Li, Ziming Wang, Sheng Shu, Wei Tang

**Affiliations:** 1 Center on Nanoenergy Research, School of Physical Science and Technology, Guangxi University, Nanning 530004, China; lichengyu@st.gxu.edu.cn; 2 CAS Center for Excellence in Nanoscience, Beijing Institute of Nanoenergy and Nanosystems, Chinese Academy of Sciences, Beijing 100083, China; wangziming@binn.cas.cn (Z.W.); shusheng@binn.cas.cn (S.S.); 3 School of Nanoscience and Technology, University of Chinese Academy of Sciences, Beijing 100049, China; 4 Institute of Applied Nanotechnology, Jiaxing 314031, China

**Keywords:** self-powered, triboelectric nanogenerator (TENG), three-group electrodes, freestanding electrode, phase difference

## Abstract

Recently, grating-structured triboelectric nanogenerators (TENG) operating in freestanding mode have been the subject of intensive research. However, standard TENGs based on interdigital electrode structures are unable to realize real-time sensing of the direction of the freestanding electrode movement. Here, a newly designed TENG, consisting of one group of grating freestanding electrodes and three groups of interdigitated induction electrodes with the identical period, has been demonstrated as a self-powered vector angle/displacement sensor (SPVS), capable of distinguishing the real-time direction of the freestanding electrode displacement. Thanks to the unique coupling effect between triboelectrification and electrostatic induction, periodic alternating voltage signals are generated in response to the rotation/sliding movement of the top freestanding electrodes on the bottom electrodes. The output peak-to-peak voltage of the SPVS can reach as high as 300 V at the rotation rate of 48 rpm and at the sliding velocity of 0.1 m/s, respectively. The resolution of the sensor reaches 8°/5 mm and can be further enhanced by decreasing the width of the electrodes. This present work not only demonstrates a novel method for angle/displacement detection but also greatly expands the applicability of TENG as self-powered vector sensors.

## 1. Introduction

Ever since TENG was proposed by Wang’s group in 2012, it has been developed rapidly nowadays [1,2,3]. Originating from the first of Maxwell’s equations, the TENG can serve as a sustainable power supply and developed four different working modes [3,4], including vertical contact-separation mode [5], lateral sliding mode [6], single-electrode mode [7], and freestanding mode [8]. Owing to its unique working principle and significant advantages of high efficiency, low cost, reliable robustness, and environmental-friendly, it is widely used in wearable electronics, energy harvesting, and self-powered sensing [9,10,11,12].

Among these four modes mentioned above, nowadays, the freestanding mode of TENG has been commonly applied in the fields of sensing and energy harvesting due to its excellent output performance, reliable robustness, and high efficiency, such as harvesting ocean wave energy, mechanical vibration, and sensing of human motions [13,14,15,16,17,18]. Lai et al. devised a single-thread-based wearable and stretchable sensor [19], and Dong et al. designed a versatile core-sheath yarn sensor [20]. Both sensors can generate signals representing the movement of joints, however, lacking of precise resolution. Afterwards, various types of TENG sensors based on freestanding mode for quantitative sensing is also reported, mainly composed of two or more grating-structured TENG [7,14,21,22,23]. Wang et al. reported a high precision self-powered angle sensor [14] and Pu et al. designed a TENG-based finger-wearable sensor [22], which both used two separate grating-structured TENG to obtain vector motion information. However, these sensing methods consume up more areas for the extra phase-referenced TENG [24].

In this work, we proposed a self-powered vector angle/displacement sensor (SPVS), which consists of a grating-segmented freestanding triboelectric layer and three groups of interdigitated electrodes with the same period. Compared to other TENG sensors, SPVS fabrication is facile, and this approach can be applied in both transverse and rotating motions [25,26]. Two grid-shaped SPVS were designed for sensing characterization because the strip and the disc grating-structured TENG have their own advantages and adaptation occasions, respectively. For the strip-shaped SPVS, the length and width of each TENG unit: 0.9 cm × 1.5 cm, while the freestanding electrode’s linear velocity is 0.1 m/s at an acceleration of 1 m/s^2^, the output voltage can reach 125 V/mm, and the minimum resolution is 3 mm. For the disc-shaped SPVS (The size of PCB is 6.2 cm in diameter), as the rotational rate ranges from 24 to 192 rpm, the voltage can reach 30 to 80 V/° and the corresponding minimum resolution is 8°. Notably, the minimum resolution of this grid structure can be further improved by reducing the width of the electrode. In all, this present work demonstrates an active vector angle/displacement sensor composed of a newly designed three-group electrode grating-structured freestanding nanogenerator, which greatly expanded TENG’s accessibility and applicability in sensing applications due to its facile fabrication process, excellent output performance, and vector characteristics.

## 2. Materials and Methods

### 2.1. Design and Fabrication of the SPVS

**Figure 1:**
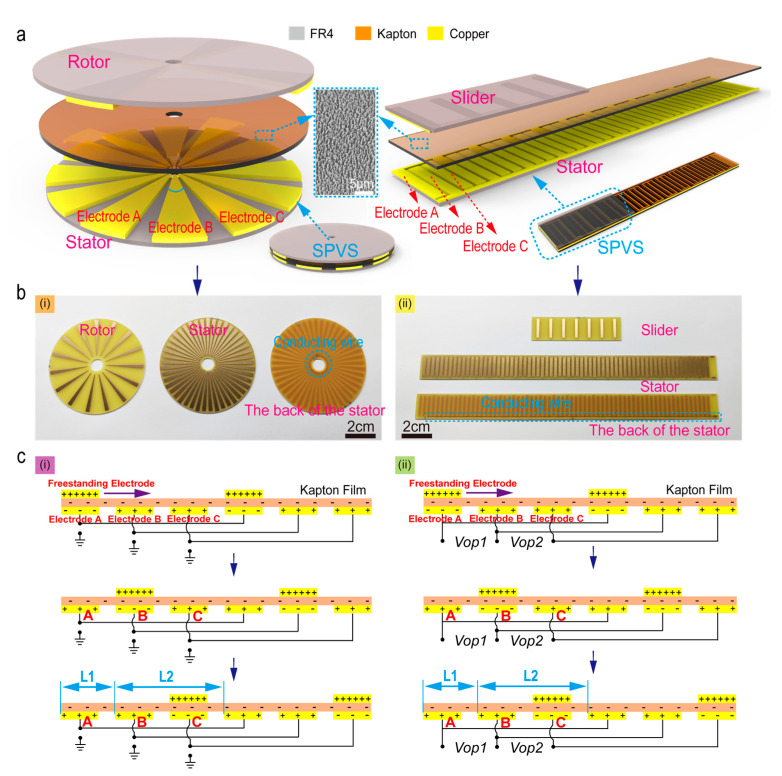
Structural design and working mechanism of the self-powered vector angle/displacement sensor (SPVS). (**a**) Schematic illustrations of disc-shaped (**left**) and strip-shaped (**right**) SPVS as well as the corresponding enlarged view of rotor/slider and stator. Inset: a scanning electron microscopy (SEM) image of the etched nanorods on the surface of Kapton film (the scale bar: 5 μm). (**b**) Optical photos of disc-shaped and strip-shaped SPVS, where a set of interdigital electrodes are connected together by conducting wires on their bottom stator. (**c**) Schematic operating principle of SPVS with two different detect modes.

The fabrication of SPVS is mainly based on the current mature printed circuit board (PCB) technology [16]. As schematically illustrated in the left of Figure 1a (SPVS with a disc structure), the upper and lower PCBs are installed coaxially. The upper disc is the rotor, and the bottom disc is the stator. Similarly, as for the strip-shaped SPVS (the right of Figure 1a), the upper and lower strips of the PCB are the slider and the stator, respectively.

The Kapton film (~35 μm thick) is employed as the dielectric triboelectric materials. It has been etched by ICP (inductively coupled plasma etching system) to achieve a rough surface and then attached to the stator’s surface to enhance the output performance of the SPVS [27]. In the ICP etching process, the Kapton film’s surface is rinsed with alcohol and deionized water and then dried with nitrogen gas. The reaction gas of argon, oxygen, and carbon tetrafluoride is introduced into the ICP chamber at flow rates of 15, 10, and 30 sccm, respectively, and etched by ICP for about 300 s (RF power is 100 W and ICP power is 400 W), and finally, Kapton film with nanorod structure was fabricated. The insert in Figure 1a represents the scanning electron microscopy (SEM) image of the etched nanostructures on the Kapton surface, and the scale bar is 5 mm. Besides, the Kapton film covered in the stator is visible in Figure S1.

Figure 1(bi,ii) depict the PCB optical photographs of SPVS with two different shaped structures, respectively, where a set of interdigital electrodes on the bottom stators are connected together by conducting wires. Moreover, the surface of the PCB substrate is embedded with periodic copper pattern gratings, and the copper (~35 μm thick) is plated with a layer of nickel-phosphorus alloy on the surface by electroless nickel/immersion gold to prevent the copper on the PCB surface from being oxidized or corroded, thereby enhancing the conductivity of the PCB circuit board. The base material of PCB is made of stiff woven glass-reinforced epoxy resin (FR4). For SPVS with a disc structure, the central angle corresponding to the electrodes A, B, and C is 5° and the gap between the bottom electrodes is 3°, and therefore, the minimum resolution is equal to 8°. For the strip-based structure SPVS, the width of the electrodes A, B, and C is 2 mm and the gap is 1 mm, and thus, the minimum resolution is 3 mm. The corresponding circuit schematic diagram of SPVS can be seen in Figure S2.

### 2.2. The Working Principle of SPVS

The working principle of SPVS is shown in Figure 1(ci,ii), representing two different measuring methods, respectively. Figure 1(ci) shows a connection that each bottom electrode is connected out to the measuring equipment, with the other end of the measuring equipment grounded. Another detection method is displayed in Figure 1(bii), where one of the bottom electrodes serves as a common end, e.g., electrode B, and thus electrode A and C acts as two output ends, forming two output voltage ports (*Vop*1 and *Vop*2).

As the top freestanding electrode (rotor/slider) rotates/slides along the surface of the Kapton film, contact electrification-induced charges will be generated and accumulated on the surface of the two triboelectric layer materials (metal copper electrode and Kapton film) [28,29]. In the initial stage, since the coupling effect of triboelectrification and electrostatic induction, the surface of the bottom electrode A overlapping with the top freestanding electrode will be negatively charged [30]. In contrast, the non-overlapped electrode (bottom metal electrodes B and C) will be positively charged. Subsequently, the bottom electrode A/B/C (stator) surface will alternately induce charges to bring about potential outside with the freestanding layer’s electrodes continuously rotate/slide, and then several groups of voltage signals with a fixed phase difference obtained through different ways of connecting the bottom electrode.

For the first connection method (the bottom electrode is grounded), as the rotor slides to the left or right, free electrons keep flowing from electrode A/B/C to the ground until the rotor returns to its initial state, forming three groups of voltage signals with a fixed phase difference. For the second connection method, free electrons keep flowing from electrode A to electrode B (or from electrode B to electrode C) until the rotor reaches the final state where the charge density on both electrodes is reversed in polarity compared with the initial state. Similarly, in this detection way, two groups of voltage signals with a fixed phase difference can also be formed. Accordingly, we can achieve real-time sensing of motion information by analyzing this analog signal in these methods, such as sensing of movement direction, sliding displacement (or rotation angle), and speed.

## 3. Result and Discussion

### 3.1. FEA Potential Simulation of SPVS

**Figure 2:**
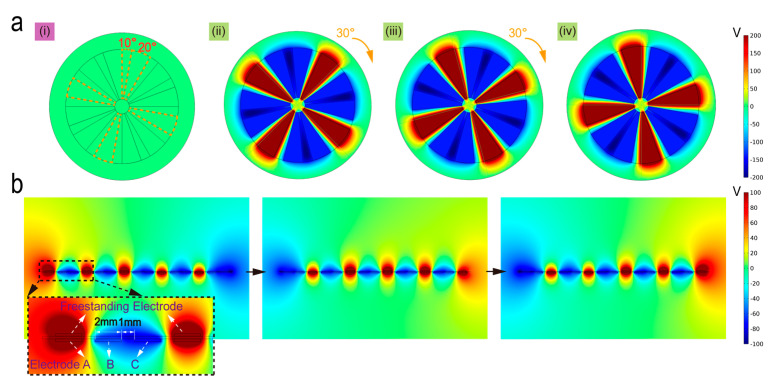
COMSOL simulation of the SPVS with different structure mode. (**a**) Potential simulation of disc structure (**i**), rotating from the initial state (**ii**) to final state (**iv**) under the rotation step of 30°. (**b**) Potential simulation of strip structure under three different states. The inset picture illustrates the position relationship between the freestanding electrode and the bottom electrodes.

Figure 2 illustrates the finite element analysis (FEA, by COMSOL Multiphysics 5.4, Stockholm, Sweden) results of the open-circuit potential distribution on the disc-shaped and strip-shaped SPVS. As depicted in Figure 2a, for the research convenience and simplicity, we set the gap between each group of electrodes at the bottom to be 10° and the central angle of the rotor to be 20° in the simulation model (the dashed line in Figure 2(ai)). With the rotor’s rotation from the initial position to the final position under a given gradient of 30° in radian, the electric potential distribution diagram can be obtained in Figure 2(ai–iv).

Similarly, as shown in Figure 2b, there are five TENG units in the simulation model, where we set each TENG unit of the width of the electrodes and the gap between the bottom three electrodes (stator) to be 2 and 1 mm, respectively. By sliding 24 mm of the slider from the initial position to the final position under a given step length of 1 mm, the diagrams of three potential distribution states can be provided in Figure 2b. The inserted enlarged image indicates the positional relationship between the top freestanding electrode as well as the bottom electrode A/B/C at the initial position.

### 3.2. The Simulation Results of SPVS

**Figure 3:**
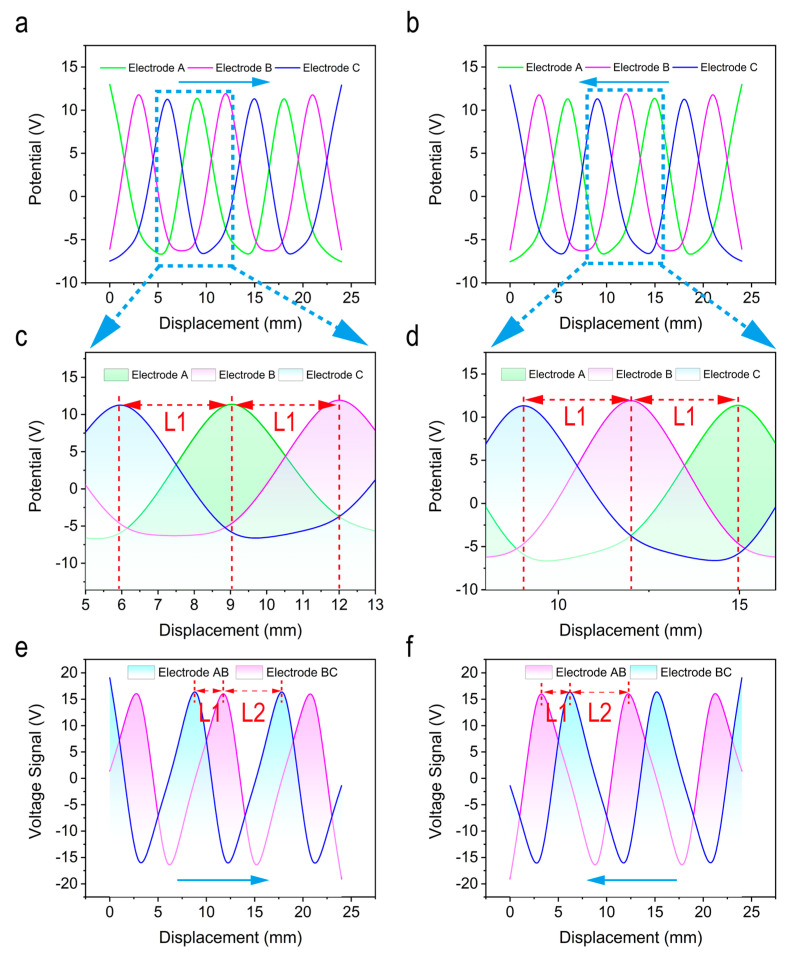
Simulation results of two detection modes of SPVS. (**a**) The theoretical calculation result of the slider sliding from left to right and (**b**) right to left under the first connection mode. (**c**,**d**) The enlarged view of the corresponding output signals in different directions. (**e**,**f**) Theoretical calculation results of the slider sliding from left to right and from right to left, respectively, under the second connection mode.

Figure 3a–f shows the simulation results of two different connection modes of SPVS. Since the simulation results of disc-shaped and strip-shaped are similar, the strip-shaped SPVS are taken as an example for further analysis. To better understand the variation of electrode potential on each TENG unit, three points on the surface of the bottom three groups electrodes were marked to obtain the potential distribution curve of the strip-shaped SPVS using the method of parametric scanning.

For the first connection method (the bottom electrode is grounded), as shown in Figure 3a,b, variations on potential curves of the bottom three groups of electrodes can be induced as the slider (the freestanding electrode) slides 24 mm in different sliding directions. However, since there is an immutable physical distance *L*1 between the bottom three group electrodes of SPVS, three sets of output potential curves with a phase difference will be acquired. The corresponding enlarged images in Figure 3c,d can vividly demonstrate the phase relationship between the three groups of output potential signals as the slider slides in different directions. To be specific, each set of output voltage curves has a phase difference of *L*1, which is precisely equal to the distance between two adjacent bottom electrodes and the distance of peaks between each potential curve. Besides, similar results are capable of being given while the slider slides in the opposite direction.

As for the second detection method, there exists a fixed phase difference between the bottom electrodes AB and BC, and thus the output voltage signals will have a phase difference of *L*1. Figure 3e,f, exhibits the simulated output voltage signals of the slider under different sliding directions, respectively, which is consistent with the theoretical analysis.

### 3.3. Strip-Shaped SPVS as a Vector Sensor

**Figure 4:**
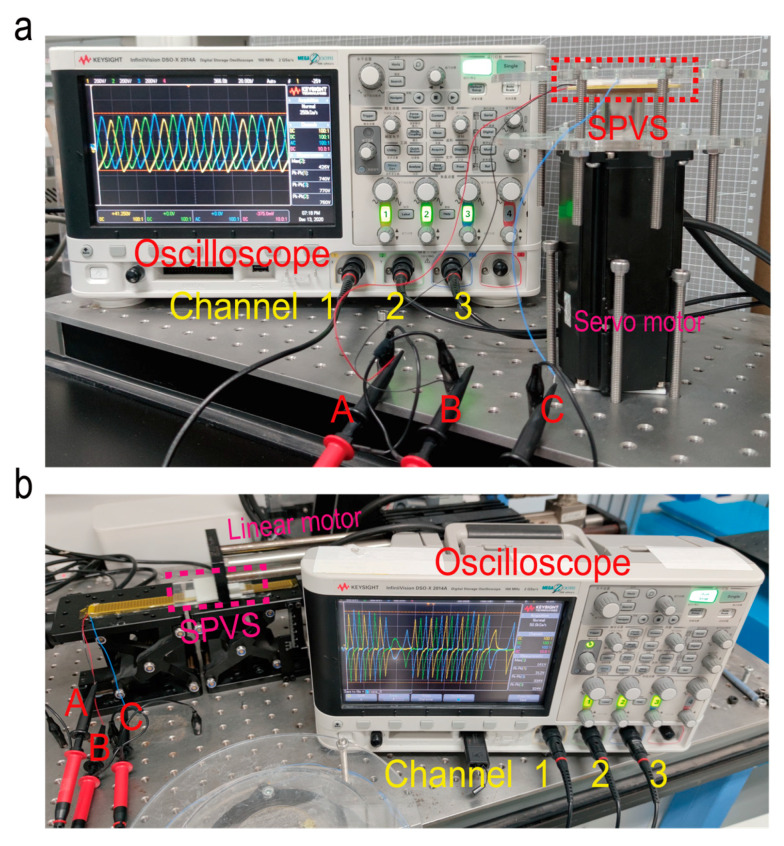
Photograph of the experimental setup for (**a**) disc-shaped SPVS and (**b**) strip-shaped SPVS.

According to the simulation results mentioned above, an oscilloscope (type: DSO2014A Keysight, Santa Rosa, CA, USA) has been employed for the experimental measurement. The experiment setup is shown in Figure 4, respectively, for the testing of disc-shaped SPVS (Figure 4a) and strip-shaped SPVS (Figure 4b).

**Figure 5:**
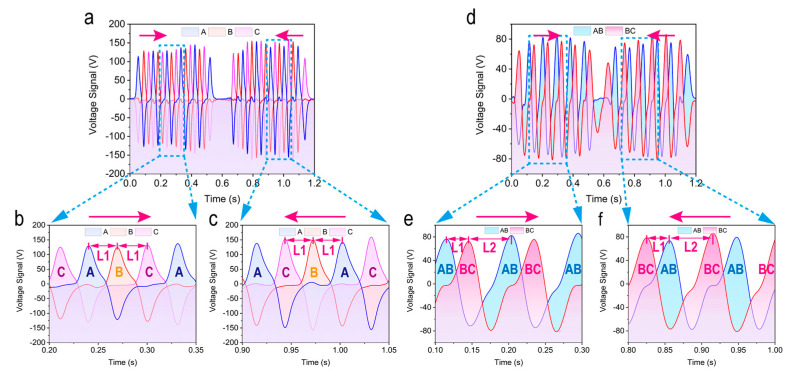
Characterizing the performance of strip-shaped SPVS. (**a**) Electrical performance of SPVS as the slider slide to the left (**b**) and (**c**) to the right under the first detection mode. (**d**) Electrical performance of SPVS as the slider slide to the left (**e**) and (**f**) to the right under the second detection mode.

Taking the measured results of strip-shaped SPVS as an example (measured by a linear motor), as shown in Figure 5a,b, when the slider slides from left to right, three sets of voltage signals with a fixed phase difference *L*1 can be continuously generated from the bottom metal electrodes A/B/C (the first detection method is utilized), which can be treated as “AB, BC, and CA,” where A, B, and C, respectively, represent the output voltage waveform curve of electrodes A, B, and C. The peak-to-peak voltage of the strip-shaped SPVS is highly about 300 V at the sliding velocity of 0.1 m/s, which the waveform of the voltage signal is similar to the superposition of triangular waves and square waves, indicating identical to the previous work and theoretical analysis. Subsequently, as the slider slides from right to left (Figure 5a,c), the bottom metal electrode A/B/C will also induct three sets of voltage signals in real-time, namely, “CB, BA, and AC.” Following the method mentioned above, one of the six waveforms in those mentioned above will appear once the slider’s sliding displacement is greater than the minimum resolution *L*1. Besides, the sliding displacement *L* of the slider can also be calculated by counting the total peak number of waveform *N* and substituting it into the formula *L* = *N* × *L*1, where *N* can be obtained by peak-counting algorithm (including the functions of filtering and derivation).

For another connection method, two different channels on the bottom metal electrodes A and B and electrodes B and C are connected to the oscilloscope (B is connected to the negative terminal) to form voltage output ports *Vop*1 and *Vop*2, respectively. As illustrated in Figure 5d,e–f, showing the electrical output performance of strip-shaped SPVS under this connection way. Due to the different physical distances between the two adjacent electrodes at the bottom, there will also generate two sets of voltage signals continuously with a fixed phase difference equal to *L*1 as the slider slides from left to right or from right to left. Moreover, similar to the first detection method for displacement calculation, for the second connection method, the sliding displacement *L* of the slider can be obtained by calculating the total number of wave peaks of *Vop*1 (or *Vop*2) *N* and substituting it into the formula *L* = *N* × (*L*1 + *L*2) = 3*L*1, where *L*2 = 2*L*1.

It is worth noting that for the SPVS based on the disc shape, the above two methods are also applicable, except that *L*1 represents the arc length of rotor rotation rather than the linear displacement. Furthermore, based on the above analysis, we can conclude that the resolution of the SPVS using the first connection method is higher than that of the SPVS using the second connection method. However, compared with the first connection method, the SPVS’s signal processing algorithm of the second connection method is simpler because only two sets of signals are involved.

### 3.4. Disc-Shaped SPVS as Vector Sensor

**Figure 6:**
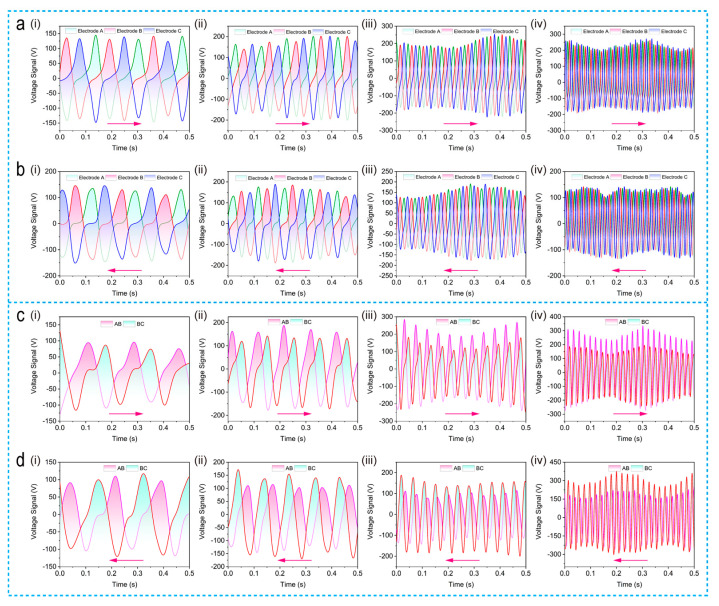
Measurement results of SPVSs’ electrical outputs working in different connection mode. (**a**), (**b**) Output voltage of disc-shaped SPVS at clockwise and anti-clockwise rotation speed of 24, 48, 96, and 192 rpm, respectively, under the first connection mode. (**c**), (**d**) Output voltage of disc-shaped SPVS at clockwise and anti-clockwise rotation speed of 24, 48, 96, and 192 rpm, respectively, under the second connection mode.

Figure 6 depicts the servo motor measured results of SPVS, since the electrical performance of the disc-shaped/the strip-shaped SPVS is similar, taking the results of disc-shaped SPVS as an example. For the first mode of three group electrode grounding detection, the rotor rotates clockwise relative to the stator at the rotation rate of 24, 48, 96, and 192 rpm, respectively, and the corresponding measured results are available in Figure 6(ai–iv), exhibiting the peak-to-peak voltage value reach as high as 400 V with a significant phase relationship after a comprehensive optimization. Similarly, three sets of output voltage curves with different phases can be obtained with the rotor rotating anti-clockwise at the same rotation rate mentioned above (Figure 6(bi–iv)). It is noteworthy that the results of sensing vector characteristics demonstrate that the output peak-to-peak voltage value could reach high of 400 V, showing the advantage and feasibility of constructing a self-powered vector measurement sensor based on TENG technology [31].

Based on the second detection mode of disc-shaped SPVS, the measured output voltage results are depicted in Figure 6(ci–iv), representing the electrical performance of disc-shaped SPVS at a clockwise rotation speed of 24, 48, 96, and 192 rpm. Similarly, as shown in Figure 6(di–iv), when the rotor rotates counterclockwise relative to the stator, the voltage curves can also be obtained at different rotation rates from 24 to 192 rpm. The peak-to-peak voltage of this connection mode can reach about 500 V with a relatively less triboelectric area, consistent with the simulation result and further verifying the TENG-based self-powered vector sensor’s feasibility.

## 4. Conclusions

This work proposed a novel designed self-powered vector angle/displacement sensor (SPVS) based on freestanding-mode TENG, consisting of three sets of interdigitated electrodes with an identical period. By detecting the phase difference of output voltage from different channels and calculating the total number of wave peaks, the active sensor based on granting-structured TENG can realize real-time monitoring and sensing of vector motion information of rotor/slider, such as angle/displacement and direction of movement [14,20]. Not only the mentioned above but we also proposed two quite efficient detection methods, both of which could be applied as vector angle/displacement sensors alone.

Furthermore, compared with other angles/displacement sensors based on TENG, the novel SPVS based on three-sets grating-structured TENG primarily has the advantage that without lowering the output voltage and increasing the area of the triboelectric layer, the SPVS could be applied for quantitative analysis of the movement with vector characteristic. In addition, the resolution of SPVS reaches 8°/5 mm and simultaneously can be further improved by decreasing the width of electrodes. In addition, using advanced PCB processing technology, SPVS will be stable facile to manufacture and low in cost, making it suitable for large-scale popularization. Furthermore, the SPVS of disc-shaped can be utilized on real-time sensing of joint motion, water flow and mechanical bearing, etc. and strip-shaped SPVS as a self-powered stretchable sensor, displacement sensor, and droplet sensor, etc. Totally, the newly designed grating-structured TENG serving as a self-powered vector angle/displacement sensor has largely expanded TENG sensors’ accessibility and applicability due to its unique design, facile fabrication, excellent output performance, and vector characteristics.

## Supplementary Materials

The following are available online at https://www.mdpi.com/2072-666X/12/3/231/s1, Figure S1: Kapton film with a thickness of around 35 μm is adhesive and can be attached to the stator as a triboelectric layer, Figure S2: The circuit schematic diagram of SPVS.
