# Supplementary material for: A Self-Powered Vector Angle/Displacement Sensor Based on Triboelectric Nanogenerator
Source: Micromachines (Basel). 2021 Feb 25;12(3):231. doi: 10.3390/mi12030231 (PMC7996610; doi:10.3390/mi12030231)
Supplement: Supplementary file 1 [file micromachines-12-00231-s001.pdf]

A self-powered vector angle/displacement sensor based on triboelectric nanogenerator

Chengyu Li, Ziming Wang, Sheng Shu and Wei Tang

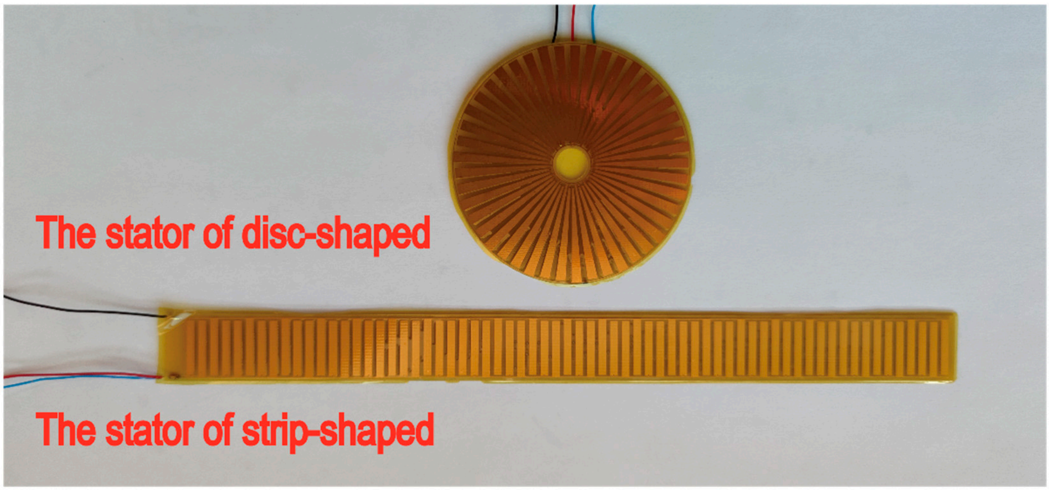

Figure S1. Kapton film with a thickness of around 35 μm is adhesive and can be attached to the stator as a triboelectric layer.

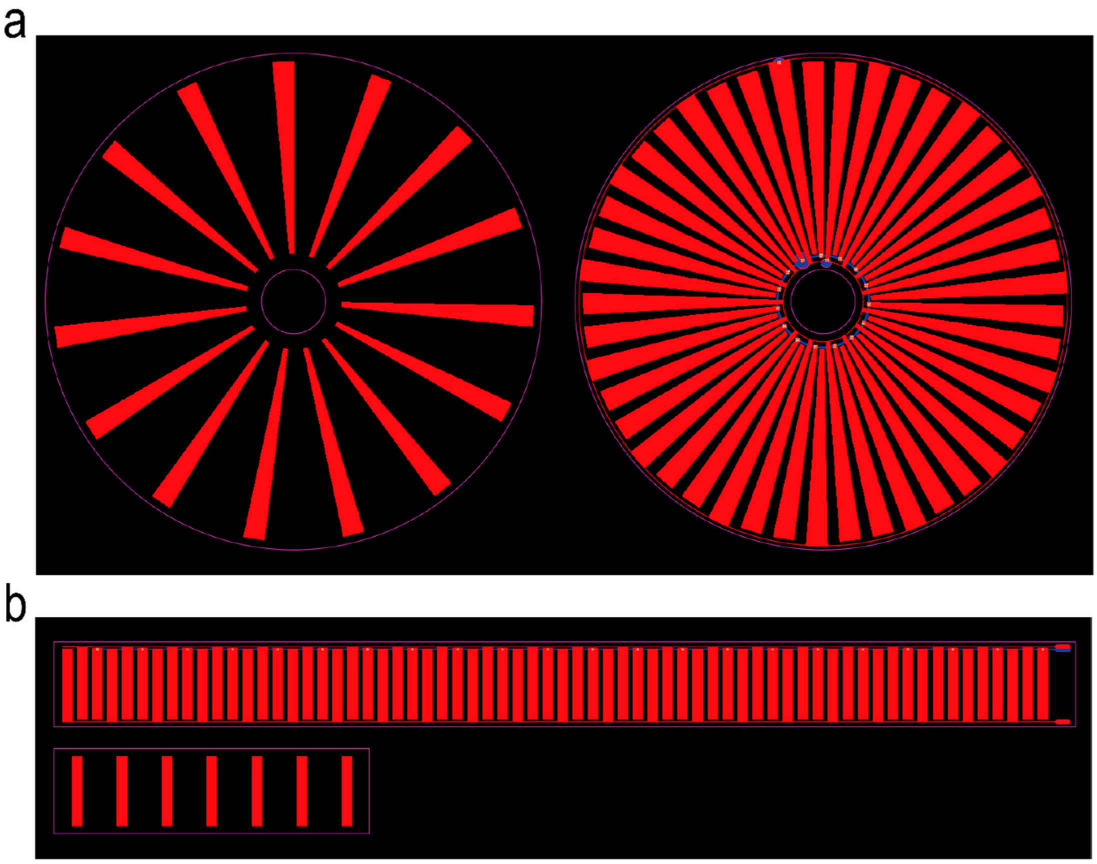

Figure S2. The circuit schematic diagram of SPVS. (**a**) PCB structural diagram based on disc-shaped SPVS as well as (**b**) strip-shaped SPVS, which red represents the front side of the PCB (the top layer), blue represents the back side of the PCB (the bottom layer), and Kapton film is attached to the top

layer of the stator PCB, generating electricity in contact with the top layer of the rotor PCB (Designed by Altium Designer 2020 software).
